# The Activated SA and JA Signaling Pathways Have an Influence on flg22-Triggered Oxidative Burst and Callose Deposition

**DOI:** 10.1371/journal.pone.0088951

**Published:** 2014-02-25

**Authors:** So Young Yi, Ken Shirasu, Jae Sun Moon, Seung-Goo Lee, Suk-Yoon Kwon

**Affiliations:** 1 Plant Systems Engineering Research Center, KRIBB, Daejeon, Republic of Korea; 2 Plant Immunity Research Group, RIKEN Center for Sustainable Resource Science, Yokohama, Japan; 3 Biochemicals and Synthetic Biology Research Center, KRIBB, Daejeon, Republic of Korea; Korea University, Republic of Korea

## Abstract

The first line of defense in plants against pathogens is induced by the recognition of microbe-associated molecular patterns (MAMP). Perception of bacterial flagellin (flg22) by the pattern recognition receptor flagellin-sensing 2 (FLS2) is the best characterized MAMP response, although the underlying molecular mechanisms are not fully understood. Here we studied the relationship between salicylic acid (SA) or jasmonic acid (JA) signaling and FLS2-mediated signaling by monitoring flg22-triggered responses in known SA or JA related mutants of *Arabidopsis thaliana* (L.) Heynh. The *sid2* mutant, impaired in SA biosynthesis, had less basal *FLS2* mRNA accumulation than the wild type, which correlated with suppression of early flg22 responses such as ROS production and induction of marker genes, *WRKY29* and *FRK1*. The JA-signaling mutants, *jar1* and *coi1,* exhibited an enhanced flg22-triggered oxidative burst and more callose accumulation than the wild type, and pretreatment with SA or coronatine (COR), a structural mimic of JA-isoleucine, altered these flg22-induced responses. Nonexpressor of pathogenesis-related genes 1 (NPR1) acted downstream of SID2 and required SA-dependent priming for the enhanced flg22-triggered oxidative burst and callose deposition. Activation of JA signaling by COR pretreatment suppressed the flg22-triggered oxidative burst and callose accumulation in a coronatine insensitive 1 (COI1) dependent manner. COR had a negative effect on flg22 responses but only the flg22-triggered oxidative burst depended on SA-JA/COR signaling antagonism. Thus the activated SA and JA signaling pathways have an influence on flg22-triggered oxidative burst and callose deposition. These results may explain how SA and JA signaling are cross talked for regulation of flg22-triggered responses.

## Introduction

Current models suggest two forms of innate immunity in plants [1]. In one model, resistance is triggered by microbe-associated molecular patterns (MAMPs) and is referred to as MAMP-triggered immunity (MTI). In the second model, effector-triggered immunity (ETI), the plant response is triggered by pathogen effectors. MTI is initiated through the recognition of conserved MAMPs by specific pattern recognition receptors (PRRs) in the plant. The best-characterized MAMP is flagellin [2], [3]. Flg22 is a 22-amino acid synthetic polypeptide that corresponds to a highly conserved epitope of the *Pseudomonas aeruginosa* flagellin protein [2]. It is widely used as a proxy for flagellin in flagellin-mediated signaling in *Arabidopsis thaliana* (L.) Heynh. Flg22 is recognized by the Arabidopsis flagellin sensing 2 protein (FLS2), a leucine-rich repeat receptor kinase [4], [5]. Activity of the downstream pathways is marked by common signaling events, such as ion fluxes, protein phosphorylation cascades, accumulation of reactive oxygen species (ROS), induction of defense genes, and cell-wall reinforcement by callose deposition [6]–[8]. By contrast, effector-triggered immunity results from the highly specific, direct or indirect interaction of pathogen effectors and the products of plant *R* genes. This recognition event leads to a strong local defense response that stops pathogen growth [9].

To survive, plants have to respond rapidly and effectively to each intruder. Plant defense signal interactions, upon an intruder's attack, can be either mutually antagonistic or synergistic and are thought to further optimize the specificity of the defense response. One of the best-studied examples of defense-related signal crosstalk is the antagonistic interaction between the salicylic acid (SA) and the jasmonic acid-ethylene (JA/ET) response pathways [10]–[12]. Biotrophic and hemi-biotrophic pathogens are generally more sensitive to SA-dependent responses, whereas necrotrophic pathogens and herbivorous insects are commonly deterred by JA/ET-dependent defense [13], [14]. ET modulates SA related plant defense signaling both positively and negatively [12]: ET has synergistic effects on SA-induced expression of *PATHOGENESIS-RELATED PROTEIN 1* (*PR 1*) [15], whereas the ET-responsive transcription factor EIN3 and EIN3-LIKE1 (EIL1) attenuate SA biosynthesis by direct binding and repression of *SALICYLIC ACID INDUCTION DEFICIENT 2* (*SID2*), encoding an SA biosynthesis enzyme [16]. SA can suppress both JA biosynthesis and sensitivity [17]. However, some of the JA biosynthetic genes are positively regulated by JA, and it does not seem to be required for the SA-mediated depression of JA signaling [18]. The protein NPR1 (for NONEXPRESSOR OF PR1) plays an important role in mediating the suppressive effect of SA down streanm of JA [17], [19].

The positive and negative regulatory components of hormone pathways are potential targets for modification of hormonal crosstalk during disease and defense. Microbial pathogens have developed the ability to manipulate plant defense responses by producing phytohormones or their functional mimics [20]. For example, coronatine (COR), a structural mimic of JA-isoleucine (JA-Ile) produced by *Pseudomonas syringae* pv. *tomato* (*Pst*) bacterium, triggers the activation of JA-dependent defense responses leading to the suppression of SA-dependent defense responses [21].

Recent studies show that SA signaling is an integral part of both the MTI and ETI defense responses. Treatment with flg22 causes SA accumulation and induces expression of canonical SA-related genes, including *SID2*, enhanced disease susceptibility 5 gene (*EDS5*), *NPR1*, and *PR1* [22], [23]. Previous studies show flg22-induced SA accumulation to be dependent on *SID2*, which encodes isochorismate synthase, a SA biosynthetic enzyme [24], [25]. MAMPs have also been reported to stimulate JA and ET production [26]–[28] by up regulating genes that encode the proteins involved in JA and ET biosynthesis [29].

Several key regulatory proteins involved in SA-JA crosstalk have been identified in Arabidopsis. The major positive regulator of the SA response, NPR1, is a possible modulator of crosstalk between the SA and JA signals [19]. The cytosolic function of the NPR1 protein is important during SA-JA crosstalk [17], [30], while the nuclear function of NPR1 is important during the activation of SA-responsive genes [19]. Coronatine insensitive 1 (*COI1*) encodes an F-box protein that regulates JA-signaling by inactivating negative regulators of JA-mediated responses [31]. The *coi1* mutant exhibits enhanced expression of SA-dependent defenses and enhanced resistance to *P. syringae* [32], [33]. The SA-mediated defense pathway is sensitized in *coi1* plants, so that SA-dependent defenses are hyper-activated in response to attack by *P. syringae*. Exogenous COR also triggered re-opening of stomata that had closed during the plants' response to MAMPs; closed stomata are part of the defense response as closure should inhibit bacterial entry into the leaf [34]. Recent reports provide evidence that COR activates three NAC genes (petunia NAM and *Arabidopsis* ATAF1, ATAF2, and CUC2): the transcription factors *ANAC019, ANAC055,* and *ANAC072*. These transcription factors then inhibit SA accumulation by regulating genes involved in SA synthesis and metabolism [35]. These reports suggested that COR-triggered SA suppression may be the molecular mechanism for COR-mediated virulence in stomata, as well as in tissues local to the infection and tissues involved in the systemic response. These findings are consistent with the hypothesis that activation of JA signaling pathway negatively results SA-dependent inducible defenses.

The goal of this study was to determine how the flg22 response and SA or JA signaling are linked. Here we investigated flg22 responses in known SA or JA related mutants and have identified *SID2* as an important component of flg22-triggered oxidative burst and early response gene induction, partially through activating the accumulation of *FLS2* mRNA. Pretreatment with SA enhanced flg22 responses through NPR1 downstream of SID2. Activated JA signaling, by COR pretreatment, acts through *COI1* to suppress the flg22 induced ROS production and callose deposition downstream of *JAR1*. These findings indicated that both SA signaling and COR mediated JA signaling are critical components in regulating flg22 responses and significantly extend our understanding of the relationship between defense-related hormone signaling and flg22 responses.

## Results

### Both SA and JA signaling are involved in flg22-triggered oxidative burst

**Figure 1:**
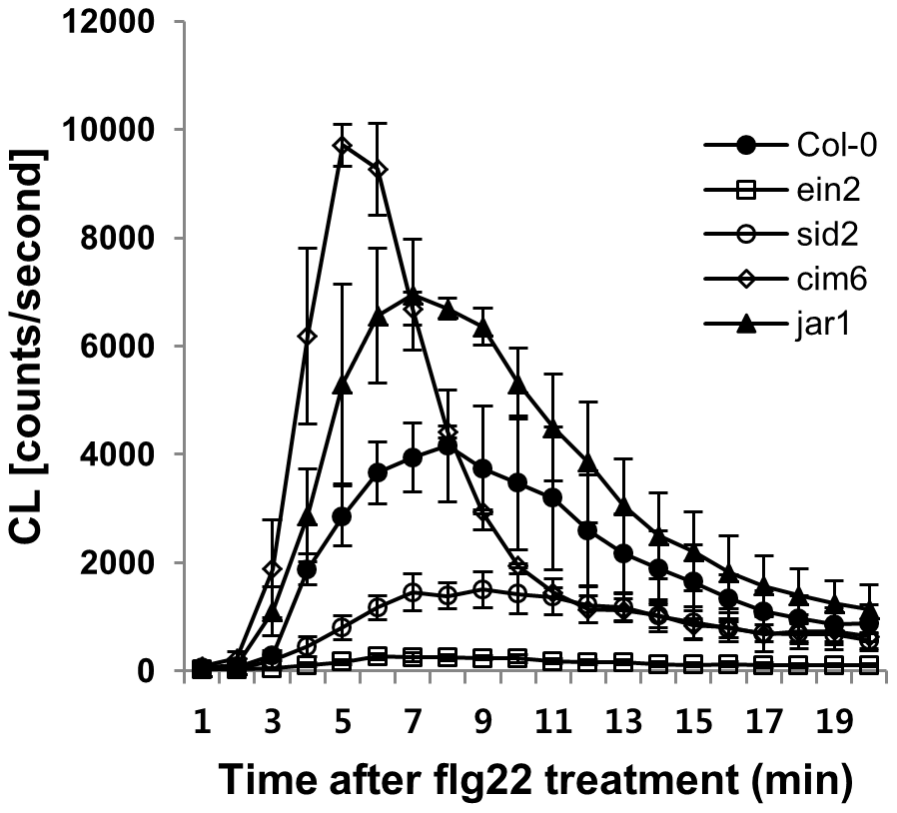
SA and JA signaling are required for flg22-triggered oxidative burst. Flg22-induced ROS generation was monitored in liquid-grown intact seedlings of indicated Arabidopsis genotypes after treatment with 1 µM flg22. Error bars represent the SD of five independent samples (n = 10) and similar results were obtained in multiple independent experiments.

One of the early reactions triggered by perception of flg22 is an oxidative burst, a rapid and transient accumulation of ROS [2]. To investigate the involvement of SA and JA in early flg22-induced responses, we monitored the flg22-triggered oxidative burst in intact seedlings of a collection of known SA- or JA-related mutants (Figs 1 and S1). The oxidative burst was diminished in the ethylene-insensitive mutant, *ein2* as described earlier [36]. In the auto-immune mutant, *cim6*, which exhibits high levels of SA accumulation and constitutive activation of SA signaling [37], the flg22-dependent ROS generation was evidently greater than that in the wild type (Fig. 1). By contrast, in *sid2* and *eds5* (also known as *sid1*) mutants [38], [39], which do not accumulate SA after either biotic or abiotic stresses, the oxidative burst was much smaller than in the wild-type (Figs 1 and S1). A clear increase in ROS production was detected in *jar1* and *fad7/fad8* mutants, which have impaired JA-signaling (Figs 1 and S1) [40], [41]. These findings indicate that the SA and JA signaling pathways are antagonistically regulated the flg22-triggered oxidative burst.

### SA or COR pretreatment induce marked changes in flg22-triggered oxidative burst

**Figure 2:**
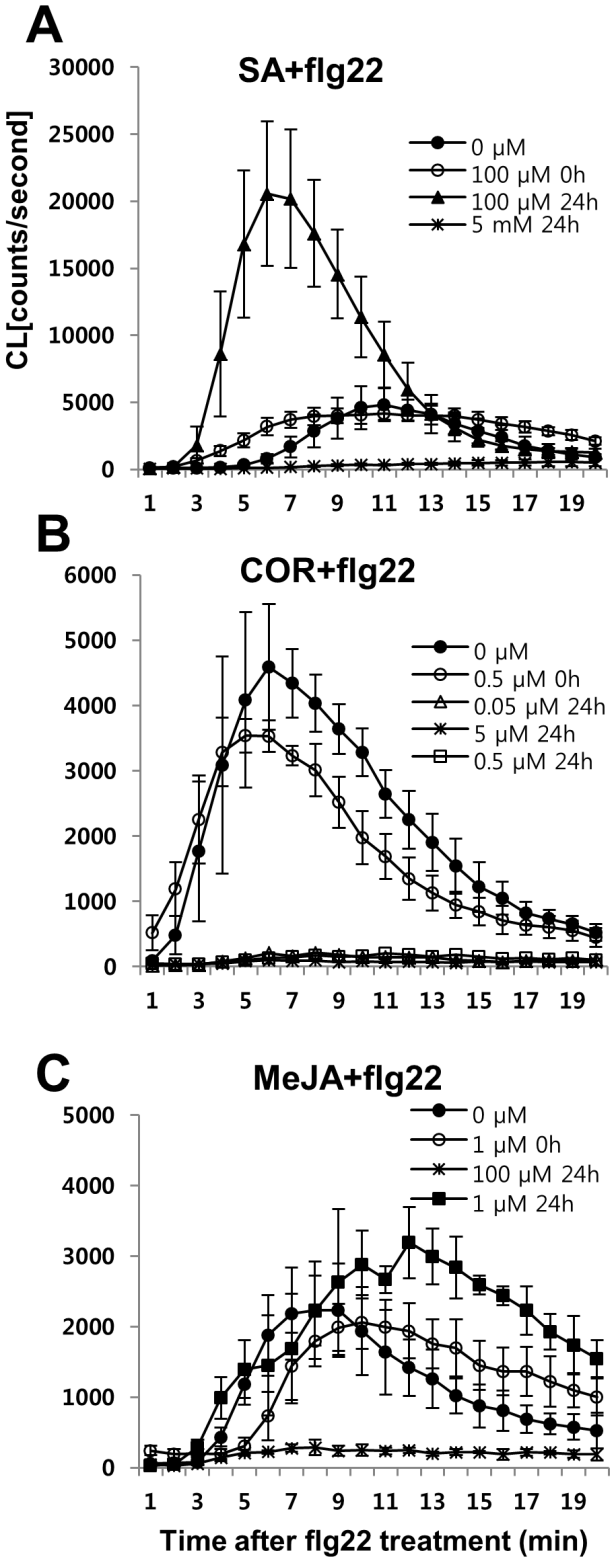
Effect of exogenous chemical treatments (SA, MeJA, or COR) on the flg22-triggered oxidative burst. (A-C) Arabidopsis seedlings were pre-incubated with various concentrations of chemicals for the indicated time periods (0 and 24 h) before the start of ROS measurements. Flg22 (1 µM) was added at zero time. Error bars represent the SD of five independent samples (n = 10) and similar results were obtained in three independent experiments.

To investigate whether exogenous SA or JA affects the flg22-triggered oxidative burst in Arabidopsis seedlings, we measured ROS levels in hormone-treated Arabidopsis seedlings. The effect of COR on the oxidative burst was also measured as many strains of the *P. syringae* synthesize COR, a JA-Ile mimic that suppresses flg22 responses by antagonizing SA-activated defense pathways [35], [42]. When seedlings were treated with SA, MeJA, or COR simultaneously with flg22, there was little effect on ROS accumulation compared to the control (Fig. 2). On the other hand, there was a marked enhancement in the flg22-triggered oxidative burst when seedlings were pretreated with SA for 24 h (Fig. 2A). This finding is similar to that of a previous report in parsley suspension cultures, in which pre-incubation with SA enhanced both spontaneous and elicitor-induced production of H_2_O_2_. The greatest effect in this study required pretreatment with >500µM SA for longer than 24 h [43].

**Figure 3:**
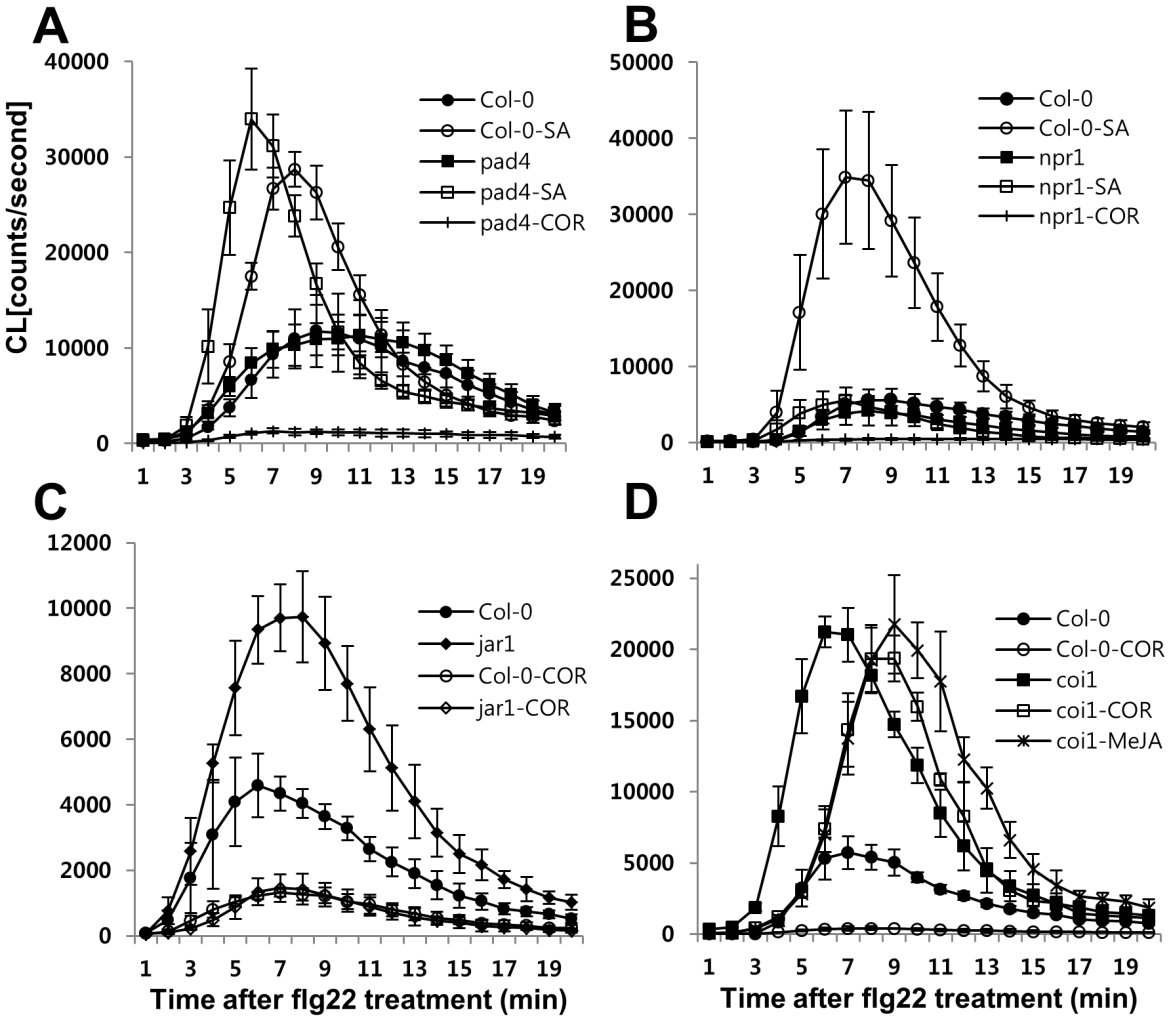
The effect of SA and COR in the flg22-triggered oxidative burst is dependent on NPR1 and COI1, respectively. (A–D) Effect of pretreatment with SA (100 µM) or COR (0.5 µM) for 24 h on the flg22-triggered oxidative burst in mutant [*pad4* (A), *npr1* (B), *jar1* (C), *coi1* (D)] and wild-type Columbia seedlings. Flg22 (1 µM) was added at zero time. Error bars represent the SD of five independent samples (n = 10) and similar results were obtained in three independent experiments.

Since the mutants *jar1, fad7/fad8,* and *coi1*, which are impaired in either JA biosynthesis or signaling, showed enhanced flg22-triggered oxidative burst as compared to the wild type (Figs 3C, 3D, and S1), we expected that exogenous MeJA and COR would reduce the burst in these mutants. Interestingly, the effect of MeJA pre-incubation on the flg22-triggered oxidative burst was relatively weak (Fig. 2C), although it was clearly suppressed by 24-h pretreatment with 0.5 µM COR and even with 0.05 µM COR (Fig. 2B). It has been suggested that COI1 directly binds to JA-Ile and COR and serves as a receptor for jasmonates [44]. Furthermore, interaction of tomato COI1 with jasmonate ZIM domain (JAZ) family proteins is highly specific for JA-Ile and structurally related JA conjugates and COR is ∼1000-fold more active than JA-Ile in promoting this interaction in vitro [45], which could explain the different results with MeJA (1 µM) or COR (0.05 µM).

To determine whether high dosages of MeJA suppress the flg22-triggered oxidative burst, we also measured ROS levels in Arabidopsis seedlings after 24 h of pre-incubation with 100 µM MeJA or 5 µM COR. As expected, the flg22-triggered oxidative burst was clearly suppressed by both chemical treatments (Figs 2B and 2C). High doses of SA (5 mM) pretreatment also obviously suppressed the flg22-triggered ROS production, which may cause indirect effects from modification of endogenous phytohormone balance (Fig. 2A). We did not detect altered ROS production by SA (5 mM), MeJA (100 µM), or COR (5 µM) pretreatment alone (data not shown). In summary, we conclude that pretreatment with low concentrations of SA enhances the flg22-triggered oxidative burst while COR or MeJA pretreatment reduces it.

### NPR1 is required for SA-mediated priming for enhancing the flg22-triggered oxidative burst; COR acts through COI1 to suppress the burst

To study the relevance of the signal component of SA in the flg22 response, we analyzed the flg22-triggered oxidative burst in the SA-signaling mutants, *pad4* [46] and *npr1* [47]. Both *pad4* and *npr1* mutants exhibited wild type like flg22-induced ROS production, while there was no SA-mediated priming effect in the *npr1* mutant compared to the wild type (Fig. 3B). This finding suggests that NPR1, but not PAD4, is required for SA-mediated priming for the enhanced flg22-triggered oxidative burst.

To investigate whether COR treatment can function as a JA-Ile mimic downstream of JAR, we measured the flg22-induced ROS level in *jar1* and *coi1*. As shown in Fig. 3C, COR still suppressed the flg22-triggered oxidative burst in *jar1*, whereas COR and MeJA were not able to suppress the burst in the *coi1* mutant (Fig. 3D). This finding indicated that COR signals act through COI1 downstream of JAR1 to suppress the flg22-induced ROS burst.

### COR compromises SA signaling-mediated priming effect on flg22-triggered oxidative burst in *cim6*

**Figure 4:**
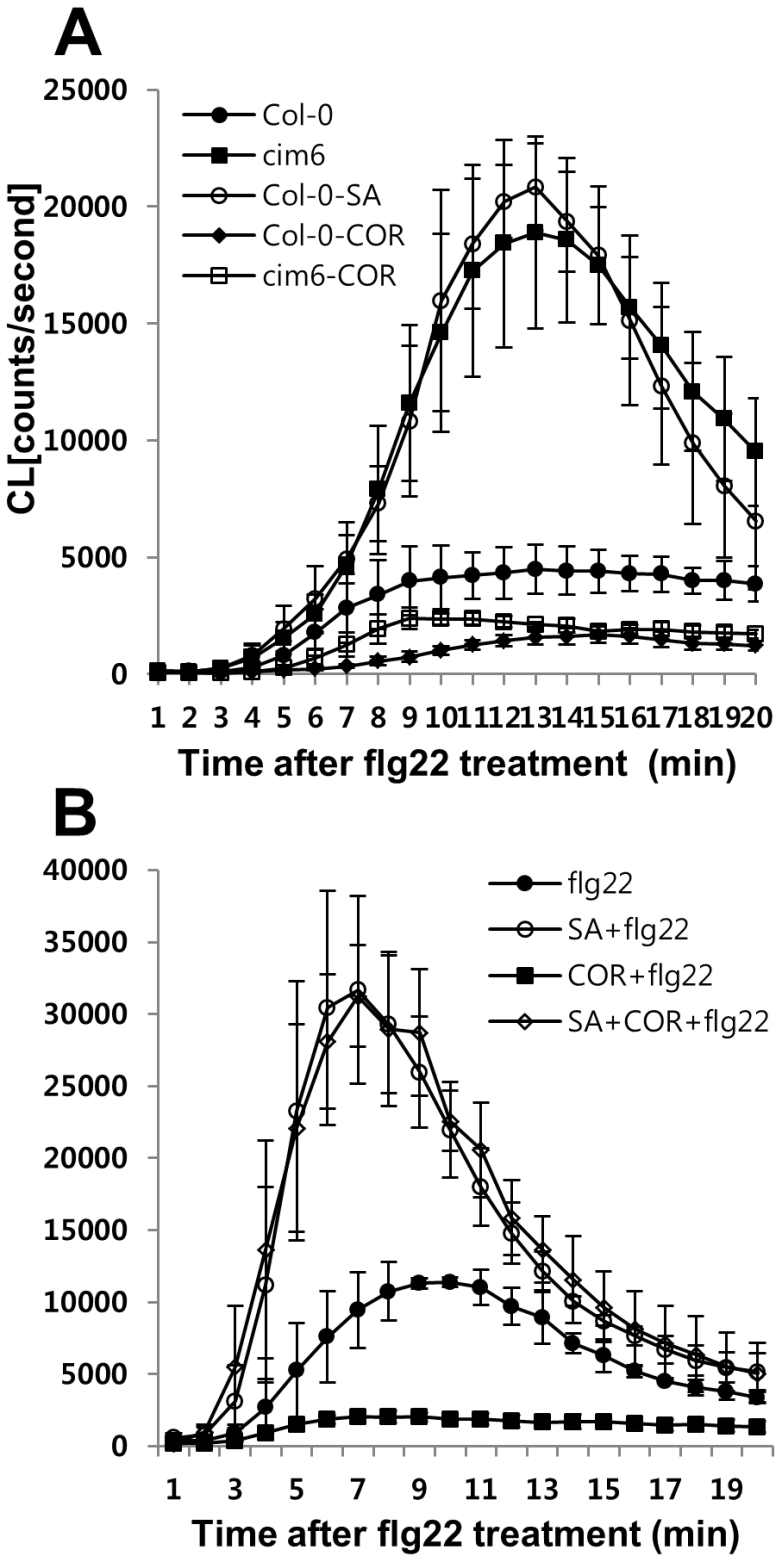
COR is required to overcome the SA effect during the flg22-triggered oxidative burst. (A) Effect of pretreatment with SA (100 µM) or COR (0.5 µM) for 24 h on the flg22- triggered oxidative burst in *cim6* and wild-type Columbia seedlings. Flg22 (1 µM) was added at zero time. (B) COR did not suppress flg22-induced ROS generation when applied simultaneously with SA. Eight-day-old seedlings were pre incubated with SA (100 µM), COR (0.5 µM), or SA plus COR for 24 h. Flg22 (1 µM) was added at zero time. Error bars represent the SD of five independent samples (n = 10) and similar results were obtained in at least two independent experiments.

To identify an association of JA-SA antagonism with the flg22-triggered ROS response, we determined if JA signaling activated by COR suppressed auto-activated SA signaling in *cim6* [37] compared to the wild type. Fig. 4A shows that the flg22-triggered oxidative burst was suppressed in *cim6* plants by pre-incubation with COR. This finding indicates that COR antagonizes activated SA signaling to suppress the flg22-triggered oxidative burst in *cim6*. When mutants seedlings were pretreated with SA and COR simultaneously, however, the SA-mediated ROS amplification was not affected by COR (Fig. 4B), suggesting that the effect of SA dominated.

### SA signaling contributes to *FLS2* transcript accumulation and early flg22 responses

**Figure 5:**
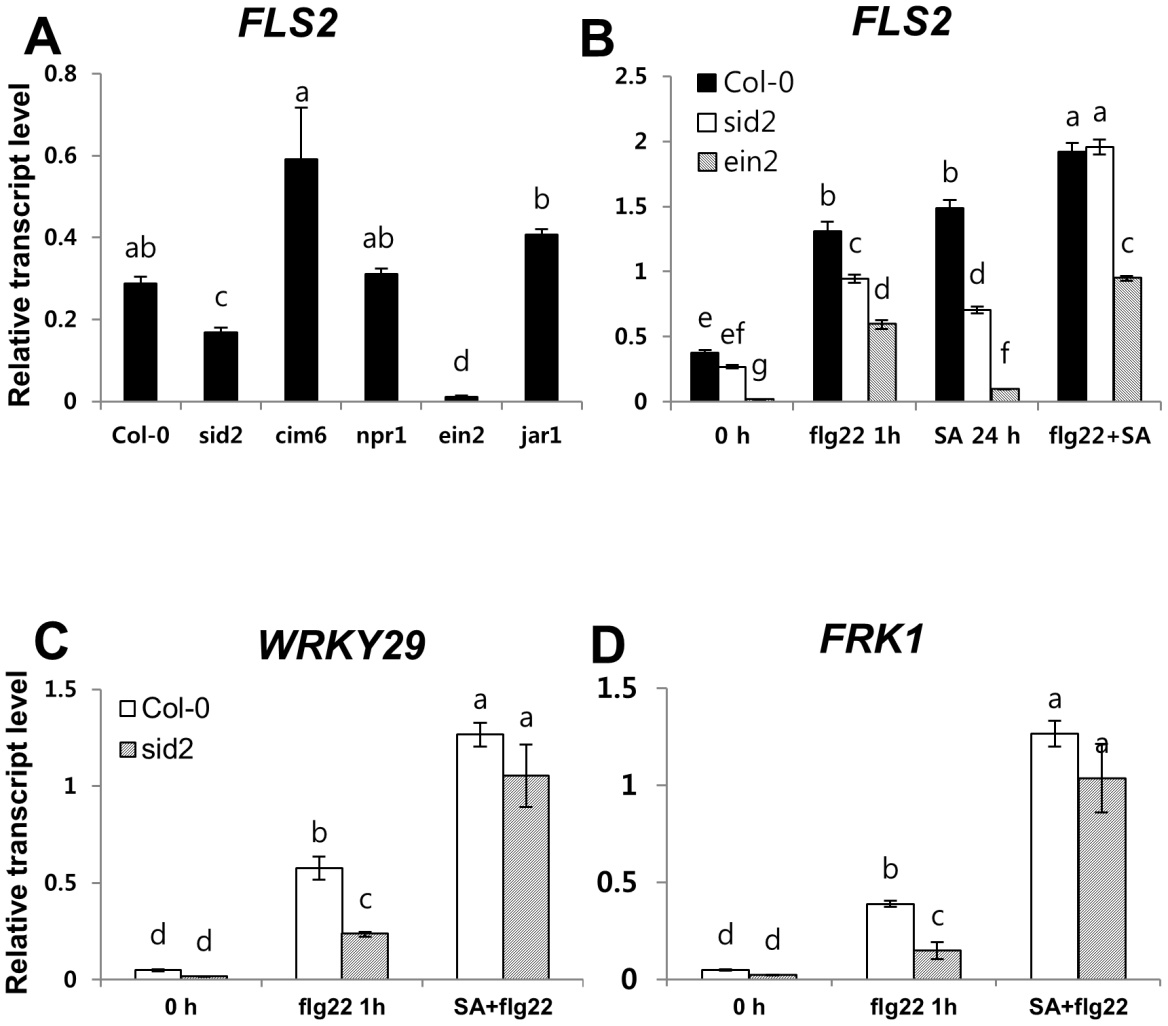
Down regulation of the flg22 response genes in *sid2* plants. For Quantitative RT-PCR analysis, 8-day-old seedlings were pre-treated with 100 µM of salicylic acid for 24 h and then incubated in 1 µM flg22 solution for 1 h. *ACT2* [74] was used as a control. Data represent SD. All quantitative gene expression measurements were performed using technical triplicate and biological duplicates. Differential letter types indicated significant differences (α = 0.05) by one-way ANOVA and Tukey HSD test of comparisons between plant genotypes with individual treatment.

*FLS2* transcript accumulation and FLS2 protein abundance affect flg22-triggered ROS generation [36]. This led us to compare transcript levels of the wild type and the mutants that were impaired in SA and JA signaling. We used the *ein2* mutant as a negative control for basal *FLS2* transcript accumulation because it is impaired in FLS2-mediated responses and these correlated with reduced *FLS2* transcription and protein accumulation [36]. The *cim6* mutant also had a high level of basal *FLS2* transcription (Fig. 5A). The *sid2* mutant is impaired in SA biosynthesis [38], and had reduced basal and flg22-induced *FLS2* transcript levels (Figs 5A and 5B). This observation was predicted, as exogenous SA alone induced *FLS2* transcript accumulation in Arabidopsis seedlings (Fig. 5B). However, the effect of *SID2* mutation on *FLS2* transcript accumulation was relatively weak when compared with that in the *ein2* (Figs 5A and 5B) indicating that SA signaling is required for full induction of *FLS2,* together with other components. These results indicate that SA signaling components play a role for *FLS2* transcript accumulation, which may affect the magnitude of the flg22-triggered oxidative burst in *cim6* and *sid2* plants.

In our study, NPR1 is required for SA-mediated priming for the enhanced flg22-triggered oxidative burst (Fig. 2A). Thus, we also analyzed SA priming effects on flg22-induced *FLS2* transcript accumulation and ROS production in *sid2* plants. SA pretreatment restored and enhanced flg22-induced *FLS2* transcription (Fig. 5B) and ROS production in *sid2* (Fig. S3). This finding indicated that NPR1 acts at SID2 downstream to regulate SA-mediated priming for enhancing the flg22-triggered oxidative burst.

Previous report showed that the mRNA levels of *WRKY29*, flg22-induced receptor-like kinase 1 (*FRK1*), and glutathion S-transferase 1 (*GST1*) were increased in Arabidopsis protoplasts within 30 min after flg22 treatment [48]. In our system, transcript levels of *WRKY29* and *FRK1* were increased in seedlings 1 h after flg22 treatment (Figs 5C and 5D) and the induction levels of *WRKY29* and *FRK1* transcripts were reduced by approximately 50% in the *sid2* mutant compared to wild-type plants (Figs 5C and 5D). SA pretreatment recovered the flg22-induced expression of *WRKY29* and *FRK1*in the *sid2* mutant to the wild-type levels (Figs 5C and 5D). These findings indicate that SA signaling involves in the regulation of the early flg22 response genes *WRKY29* and *FRK1*. In our system, SA signaling was not only required for *FLS2* mRNA accumulation but also for downstream events, including ROS production and early flg22 response gene accumulation. We suggest that SA signaling contributes to early flg22 responses through activating *FLS2* mRNA accumulation. Consistent with our results, Assai and colleagues demonstrate that flg22 signaling leading to the expression of *WRKY29* and *FRK1* requires FLS2 [48].

### SA or COR pretreatment trigger marked changes in flg22-induced callose deposition

**Figure 6:**
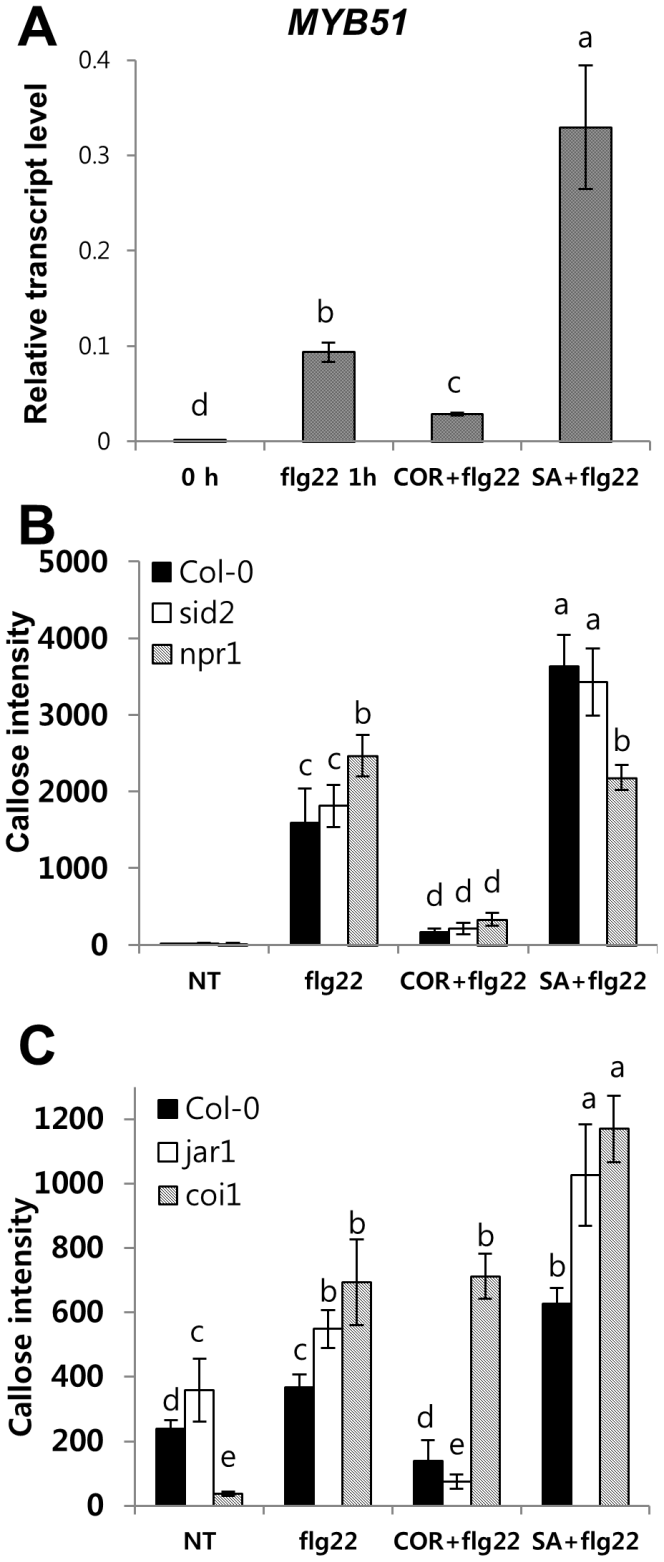
Effect of SA or COR pretreatment on flg22-induced *MYB51* transcript accumulation and callose deposition of Arabidopsis seedlings. (A) *MYB51* transcripts were measured in 8-day-old seedlings 1 h after treatment with 1 µM flg22. Data represent SD. All quantitative gene expression measurements were performed using technical triplicates and biological duplicates. (B–C) Eight-day-old seedlings were pre-incubated with SA (100 µM) or COR (0.5 µM) for 24 h, after which the seedlings treated with flg22 for 1 h were stained with aniline blue. Relative callose intensities were quantified as the number of fluorescent callose-corresponding pixels relative to the total number of pixels covering plant material. Values represent SE, n>6. Differential letter types indicated significant differences (α = 0.05) by one-way ANOVA and Tukey HSD test of comparisons between plant genotypes with individual treatment.

Another well-studied flg22-elicited response in Arabidopsis is the deposition of callose, a β (1-3)-glucan polymer, which is regulated by indole glucosinolates (IGs) [7]. MYB51 is a transcription factor essential for the regulation of IGs biosynthesis [49]. The SID2 mutation had little effect on flg22-induced expression of *MYB51* (Fig. S4) and *sid2* plant exhibited wild-type-like flg22-induced callose response. This finding indicated that MYP51 functions downstream of SID2 or a SID2-independent pathway to regulate the flg22-induced callose accumulation. However, SA or COR pretreatment markedly affected the flg22-induced *MYB51* mRNA level (Fig. 6A). COR pretreatment significantly reduced flg22-induced expression of *MYB51,* while SA pretreatment greatly enhanced its transcript abundance in cotyledons 1 h after flg22 treatment (Fig. 6A). To determine whether altered *MYB51* transcript abundance is correlated with flg22-triggered callose deposition, we measured callose deposition in COR or SA pretreated Arabidopsis cotyledons. Pretreatment with COR suppressed flg22-induced callose deposition in the wild-type and *jar1* cotyledons, but not in *coi1* (Figs 6C and S5B). This finding indicated that COR signals act through COI1 to suppress the flg22-induced callose deposition the downstream of JAR1. Pretreatment with SA enhanced flg22-induced callose deposition in all of the mutants tested except *npr1,* indicating that SA primes callose deposition through NPR1 downstream of SID2 (Figs 6B and S5A). In summary, NPR1 is required for SA-mediated priming for enhancing both flg22-induced ROS production and callose deposition, while COR suppresses flg22-induced ROS production as well as callose response through COI1 (Fig. 2B). Based on these results, we suggest that the altered flg22-triggered oxidative burst resulting from COR or SA pre-incubation might affect flg22-induced callose deposition. Actually, a model system has been used to demonstrate that ROS act as positive signals in flg22- and oligogalacturonides (OGs)-induced callose deposition [50], [51].

## Discussion

Recent studies have shown that SA signaling is an integral part of the flg22 responses. Flg22 treatments caused SA accumulation in a *SID2*-dependent manner that caused gene expression changes and pathogen growth [23]. Resistance to *Pst* DC3000 induced by pre-treatment with flg22 was compromised in *sid2* plants, demonstrating that flg22-induced SA is important for MAMP-triggered resistance [23], [52]. However, flg22-elicited bacterial resistance corresponds to a late flg22 response. Our study points to the impact of SA signaling at an early stage of the flg22 response, oxidative burst that may be involved in late flg22 response, callose deposition. Here, we found that *cim6* and *sid2* had altered *FLS2* mRNA levels, which correlated with the level of the flg22-triggerd oxidative burst. SA signaling is also involved in the regulation of the early flg22 response genes, *WRKY29* and *FRK1* (Figs 5C and 5D). These findings demonstrated that SA signaling is required for not only a late flg22 response but also for early flg22 responses. Furthermore, we provide new evidence that NPR1 is involved in SA-dependent priming for enhancing the flg22-triggered oxidative burst and callose deposition (Figs 3B and 6B). SA signaling and COI1-dependent signaling are antagonistic to one another [53]. Similarly, in our system, COR had a negative effect on flg22 responses but only the flg22-triggered oxidative burst depended on SA-JA/COR signaling antagonism. COR suppressed flg22-induced ROS production in *cim6* (Fig. 4A) while still reducing callose deposition in *sid2* (Fig. 6B). This finding suggests that, in addition to antagonizing one another, they regulate flg22-induced responses independently.

### SA signaling contributes to basal *FLS2* mRNA accumulation

*SID2*, an essential gene for SA synthesis [25] was required for the flg22-triggered oxidative burst (Fig. 1). Because the flg22-triggered oxidative burst occurred within a few minutes of elicitation, we hypothesized that SA signaling components may modulate early flg22-responses, possibly by controlling *FLS2* accumulation. To test this hypothesis, we measured the basal levels of the *FLS2* transcript in *sid2* and *cim6* by qRT-PCR analysis. Interestingly, basal *FLS2* mRNA was strongly enhanced in *cim6* and suppressed in *sid2*, compared to the wild type (Fig. 5A). However, the effect of the *SID2* mutation on *FLS2* transcript accumulation was relatively weak when compared to that in the *ein2* mutant (Figs 5A and 5B), suggesting that SA signaling components accompany other factors to regulate *FLS2* mRNA accumulation. According to a recent report, ET signaling also contributes to *FLS2* expression. EIN3 and EIN3-like transcription factors, which require EIN2 activity to accumulate, directly control *FLS2* expression [54]. Our results confirm this previous report: suppressed expression of *EIN2* in *sid2* plants before flg22 treatment (Fig. S2). In the absence of flg22, the intact *SID2* might be required for *EIN2* transcript accumulation. Importantly, *SID2* is not a classical transcription regulator and therefore, it is unlikely to regulate *EIN2* or *FLS2* gene expression directly. Further study of the mechanism of *EIN2* transcript regulation in *sid2*, including the relationship between ethylene-signaling and *EIN2* mRNA level, may reveal any *SID2* function in *FLS2* transcript regulation.

### NPR1 plays a role in SA-mediated priming for enhancing flg22 responses

Establishment of systemic acquired resistance (SAR) requires a functional SA signaling pathway and is closely associated with systemic SA accumulation and systemic expression of a set of pathogenesis-related (PR) and other defense genes [55]. Priming is a phenomenon that enables cells to respond to much lower stimulus in a more rapid and robust manner than do nonprime cells [56], [57]. An example of priming comes from studies of parsley by Kauss and Jeblick, 1995 and Thulke and Conrath, 1998. Our data also showed that pretreatment with low doses of SA strongly enhanced the flg22-triggered oxidative burst, marker gene accumulation, and callose deposition in Arabidopsis seedlings.

The *sid2* mutant plants exhibit diminished early flg22 responses while the *npr1* mutant is not defective in flg22 responses (Figs 1, 3B and 6B). The *npr1* mutant accumulates wild-type-like SA levels in response to avirulent pathogen inoculation. However, *npr1* mutants are unable to express induced SAR [47], [58]. In this study, exogenous SA served as an flg22-signaling enhancer. The *npr1* plants, however, did not show SA-dependent enhancement of the flg22-triggered oxidative burst or callose response (Figs 3B and 6B), indicating that NPR1 is involved in SA-mediated priming that enhanced flg22-induced responses (Fig. 3B). Consistent with our results, there are other reports that NPR1 plays a role in SA-mediated priming for enhanced defense responses [56], [59]. These potentiated responses suggest that the priming of defense responses is not solely confined to the SAR response. NPR1-mediated priming of defense responses also demonstrated in flg22 responses (Figs 3B and 6B).

Although the molecular basis of SA-mediated priming for enhancing flg22 responses is unclear, we hypothesize that SA pretreatment act at the post-translational level by protein modification. SA has been shown to control the nuclear translocation of NPR1 through cellular redox changes [60], [61]. NPR1 homeostasis is controlled by SA binding to NPR3/NPR4 in a concentration-dependent manner. In wild-type plants, low basal SA levels may bind to NPR4, thereby allowing some NPR1 to accumulate to confer basal resistance [62], [63]. Free stable NPR1 monomer might not be sufficient for the activation of the FLS2 downstream event that is required for the recognition of flg22 by FLS2. In our system, pre-incubation with 100 µM SA alone promoted FLS2 transcript regulation (Fig. 5B) while it did not trigger ROS production (data not shown). The enhanced level of *FLS2* mRNA and free stable NPR1, possibly due to SA pretreatment, might contribute to accelerated FLS2-dependent flg22 responses.

### How does COR signaling link the flg22-triggered responses?

Antagonism between SA and JA has been reported, mostly as SA inhibiting JA [64], although a few cases show an antagonistic relationship of JA on SA signaling. The higher SA content of the *coi1* mutant, compared to the wild type, is one example of this relationship [65]. A recent report provides evidence that COR pretreatment suppresses SA accumulation through three NAC genes: *ANAC019, ANAC055,* and *ANAC072*. These NAC transcription factors exert this inhibitory effect by repressing *SID2* (*ICS1*) and *SA methyl transferase 1* (*BSMT1*) genes involved in SA biosynthesis and metabolism, respectively [35]. In this study, COR pretreatment suppressed the enhanced flg22-triggered oxidative burst (Fig. 4A) in Arabidopsis seedlings. Furthermore, three JA-signaling mutants, *jar1*, *coi1, and fad7/fad8* were hypersensitive to flg22. The JA signaling mutants exhibit an almost three-fold increase in flg22-dependent ROS generation over the wild type. (Figs 1, S1, 3D). Based on this result, we suggest that SA signaling is required for canonical flg22-triggered ROS production and, therefore, COR-mediated suppression of the burst, representing one mechanism that underlies JA-SA antagonism.

Flg22-induced callose deposition is regulated by ROS [50], [51], miRNA signals generated by RNA interference regulatory protein Argonautel [66] and glucosinolate-derived metabolites [7]. Furthermore, SA is also involved in microbe-triggered callose deposition [7], [67], suggesting that there are multiple signaling pathways in flg22-induced callose formation. COR pretreatment inhibited flg22-induced callose deposition in both wild type and *sid2* (Fig. 6B). These results suggest that COR may function downstream of SID2 or in an SA-independent pathway to suppress flg22 induced callose response. An observation similar to ours was made in Arabisopsis roots. PAMP-induced callose deposition, which does not require SA signaling, was suppressed by COR [68]. There are also recent reports that COR suppresses an SA-independent pathway and contributes to callose deposition by reducing the accumulation of an indole glucosinolate upstream of the activity of the penetration 2 (PEN2) myrosinase [69].

### What is the role of flg22-triggered oxidative burst in late flg22 responses?

Although both Respiratory Burst Homolog proteins D and F (RbohD and RbohF) may regulate plant defense responses [70], RbohD alone was sufficient for the PAMP-triggered oxidative burst [51]. However the precise role of the flg22-triggered oxidative burst in FLS2 downstream events is unclear. Recently, Luna and associates (2011) proposed that flg22-induced callose deposition is controlled by RbohD-dependent H_2_O_2_ and that glucosinolate metabolites act downstream of RbohD-generated H_2_O_2_ in the regulation of flg22-induced callose deposition. The *rbohD* mutant is blocked in the flg22-induced callose response and flg22-induced H_2_O_2_ was also dramatically reduced in this mutant [71]. Because both the flg22-triggered oxidative burst and callose deposition are controlled by RbohD-dependent ROS [51], [71], it is probable that there is a relationship between the burst and callose response. To test whether alteration in the flg22-triggered oxidative burst is correlated with the abundance of flg22-induced callose, we measured flg22-induced callose deposition in SA-or COR-pretreated Arabidopsis cotyledons and assessed the correlation between ROS level and callose abundance. NPR1 regulates SA-induced priming for enhancing flg22-induced ROS, which correlated with enhancement of the flg22-induced callose response. Activated JA signaling by COR suppressed the flg22-triggered oxidative burst through COI1, which correlated with suppression of flg22-induced callose deposition. Based on these findings, we suggest that there is a relationship between the flg22-mediated oxidative burst and flg22-induced callose deposition. Interestingly, *sid2* plants had a lower flg22-triggered oxidative burst than the wild type, although callose accumulation was unaltered, suggesting that NPR1 acts downstream of *SID2* in the regulation of SA-mediated priming for enhanced flg22 responses. Although the molecular basis is currently unknown, an interaction between SA or JA signaling and the flg22-triggered oxidative burst seems to be required in regulation of callose deposition, a late flg22 effect. Further studies will be required to elucidate how the SA- or COR-mediated signaling acts in regulation of the flg22-triggered oxidative burst.

## Materials and Methods

### Plant growth conditions and chemical treatment

*Arabidopsis thaliana* (L.) Heynh lines used in this study were derived from the Columbia (Col) ecotype. These lines were *cim6*; CS6571, *coi1*, *ein2*; CS3071, *eds5*; CS3735, *fad7fad8*; CS8036, *jar1*; CS8072, *npr1*; CS3726, *pad4*; CS3806. The line *sid2* was provided by Ken Shirasu [72]. Seeds of *Arabidopsis* were surface-sterilized using a gas sterilization method and planted in the wells of a 48-well microtiter plate. Each well contained MGRL nutrients [73] supplemented with 0.1% sucrose. After sealing the plates with surgical tape, they were placed at 4°C for two days to break dormancy and incubated in a 16-h light/8-h dark cycle at 22°C. Exogenous chemicals were applied at the following concentrations: 1 µM flg22 (Peptron, http://www.peptron.com), 0.05–5 µM COR (Sigma-Aldrich), 0.1–1 mM SA (Sigma-Aldrich) and 0.1–100 µM MeJA (Sigma-Aldrich).

### Oxidative burst measurements

ROS were measured in eight-day-old seedlings. Seedlings were incubated in a 48-well microtiter plate containing 700 µL MGRL solution supplemented with 0.1% sucrose and 100 µM L-012 (a chemiluminescence probe; Wako, Japan). After 2 h incubation in 100 µM L-012 containing MGRL solution, 1 µM flg22 was added. A multi-label reader, VICTOR X3 (Perkin Elmer, USA), was used to verify the results we obtained from the L-012-derived chemiluminescence (CL; counts per second; cps) at 590-nm emission.

### Quantitative real-time polymerase chain reaction analysis

Total RNA was isolated from the collected seedlings using RNaesy mini kit (Qiagen) according to the manufacturer's instruction. Approximately 1 µg DNA-free RNA was used for first-strand cDNA synthesis using the Moloney Murine Leukemia Virus (M-MuLV) reverse transcriptase for quantitative real-time polymerase chain reaction (qRT-PCR; Fermentas) according to the manufacturer's instruction. The qRT-PCR reactions were performed using a Thermal Cycler Dice Real Time System TP850 (TaKaRa, http://www.takara-bio.com) and SYBR Premix Ex Taq (TaKaRa). Primer sets (final concentration of 0.1 µM for each primer) were used for a final volume of 25 µL. The thermal profile of the qRT-PCR reactions was 10 min at 95°C, 40 cycles of 5 s at 95°C/20 s at 60°C. Subsequently, a dissociation curve was generated. All reactions were carried out in triplicate. Primers used for qRT-PCR are listed in the Supporting Information.

### Aniline blue staining, microscopy analysis and callose quantification

Seedlings were collected, stored in 95% ethanol, and stained with aniline blue as described previously, with some modification [5]. Briefly, seedlings were incubated for at least 24 h in 95–100% ethanol until all tissues were transparent, washed in 0.07 M phosphate buffer (pH = 9), and incubated for 1–2 h in 0.07 M phosphate buffer containing 0.01% aniline blue (Sigma) prior to microscopic analysis. A minimum of eight cotyledons per condition per experiment were visualized under ultraviolet light with an epifluorescence microscope (Nikon AZ 100 M). Callose was selected manually, using the “magic wand” tool in Photoshop CS5. Callose-corresponding pixels and the number of depositions were recorded as the area covered by the total number of selected pixels and number of measurements, respectively, using the “record measurements” tool in Photoshop CS5. Average callose measurements were based on at least six photographs from different seedlings [71].

## Supporting Information

Table S1 **Primers for qRT-PCR analysis.**
(DOCX)

Figure S1 **SA- and JA-signaling are required for the flg22-triggered oxidative burst.** Flg22-induced ROS generation was monitored in liquid-grown intact seedlings of the indicated genotypes after treatment with 1 µM flg22. Error bars represent the SD from five independent samples (n = 10) and similar results were obtained in multiple independent experiments.
(TIF)

Figure S2 **Down regulation of the** ***ein2*** **gene in** ***sid2*** **plants.** For Quantitative RT-PCR analysis, 8-day-old seedlings were pre-treated with 100 µM of salicylic acid for 24 h and then incubated in 1 µM flg22 solution for 1 h. *ACT2* [74] was used as a control. Data represent SD. All quantitative gene expression measurements were performed using technical triplicate and biological duplicates. Differential letter types indicated significant differences (α = 0.05) by one-way ANOVA and Tukey HSD test of comparisons between plant genotypes with individual treatment.
(TIF)

Figure S3 **SA pretreatment reversed the suppressed flg22 response in** ***sid2*** **mutants.** For ROS measurement, 8-days-old seedlings were pretreated with 100 µM SA for 24 h and 1 µM flg22 was added at zero time. *ACT2* was used as control. Error bars represent the SD of five independent samples (n = 10) and similar results were obtained in three independent experiments.
(TIF)

Figure S4 **Effect of SA or COR pretreatment in flg22-induced** ***MYB51*** **mRNA accumulation.** Quantitative RT-PCR analysis of MYB51 gene expressions were measured in 8-day-old seedlings 1 h after treatment of 1 µM flg22. *ACT2* was used as control. Data represent SD. All quantitative gene expression measurements were performed using technical triplicates and biological duplicates. Differential letter types indicated significant differences (α = 0.05) by one-way ANOVA and Tukey HSD test of comparisons between plant genotypes with individual treatment.
(TIF)

Figure S5 **Effect of either SA or COR pretreatment on flg22-induced callose deposition.** At 24 h post-treatment, cotyledons were stained with aniline blue. Fluorescence was observed with a NIKON AZ 100 M microscope. Representative images shown here came from eight leaves of eight independent plants, and similar results were obtained from two independent experiments.
(TIF)
