# Supplementary material for: The Activated SA and JA Signaling Pathways Have an Influence on flg22-Triggered Oxidative Burst and Callose Deposition
Source: PLoS One. 2014 Feb 25;9(2):e88951. doi: 10.1371/journal.pone.0088951 (PMC3934882; doi:10.1371/journal.pone.0088951)
Supplement: Table S1 — Primers for qRT-PCR analysis. (DOCX) [file pone.0088951.s001.docx]

**Table S1.** Primers for qRT-PCR analysis

| **Primer name** | **Primer sequence** | **Reference** |
| --- | --- | --- |
| EIL1_F | AGCTTCAGGACACGACTCTTGGTT | (Shi *et al.,* 2012) |
| EIL1_R | TTAGGCCACCAAGGTGGAGAAACT |  |
| EIN2_F | CGCAAGCATCGTCCCTCACAATTT | (Shi*et al*., 2012) |
| EIN2_R | ACAAGTGACAGTCCGCTGAAGACA |  |
| EIN3_F | TCCTGCACCTTACAAGAAGCCTCA | (Shi *et al*., 2012) |
| EIN3_R | TTGCCTCACGAGCTTACGGATCTT |  |
| FLS2_F | ACTCTCCTCCAGGGGCTAAGGAT | (Boutrot *et al.,* 2010) |
| FLS2_R | AGCTAACAGCTCTCCAGGGATGG |  |
| FRK1_F | ACGGGCATAGTTCCACAAAG | (Trujillo *et al.,* 2008) |
| FRK1_R | CGTCAAAAGAACGACGATGA |  |
| GST1_F | AGTTTTCGGTCACCCAGCTTC | (Nakagami *et al.,* 2006) |
| GST1_R | AGAACCTTCTGAGCAGAAGGC |  |
| MYB51_F | ACAAATGGTCTGCTATAGCT | (Daudi *et al*., 2012) |
| MYB51_R | CTTGTGTGTAACTGGATCAA |  |
| WRKY29_F | GCGTAAATACGGGCAGAAAC | (Trujillo *et al*., 2008) |
| WRKY29_R | GGTTTGGGTTGGGAAGTTTT |  |
| ACT2_F | AGTGTCTGGATCGGTGGTTC | (Qiu *et al*., 2008) |
| ACT2_R | CCCCAGCTTTTTAAGCCTTT |  |
