# Supplementary figures and images for: The Activated SA and JA Signaling Pathways Have an Influence on flg22-Triggered Oxidative Burst and Callose Deposition
Source: PLoS One. 2014 Feb 25;9(2):e88951. doi: 10.1371/journal.pone.0088951 (PMC3934882; doi:10.1371/journal.pone.0088951)

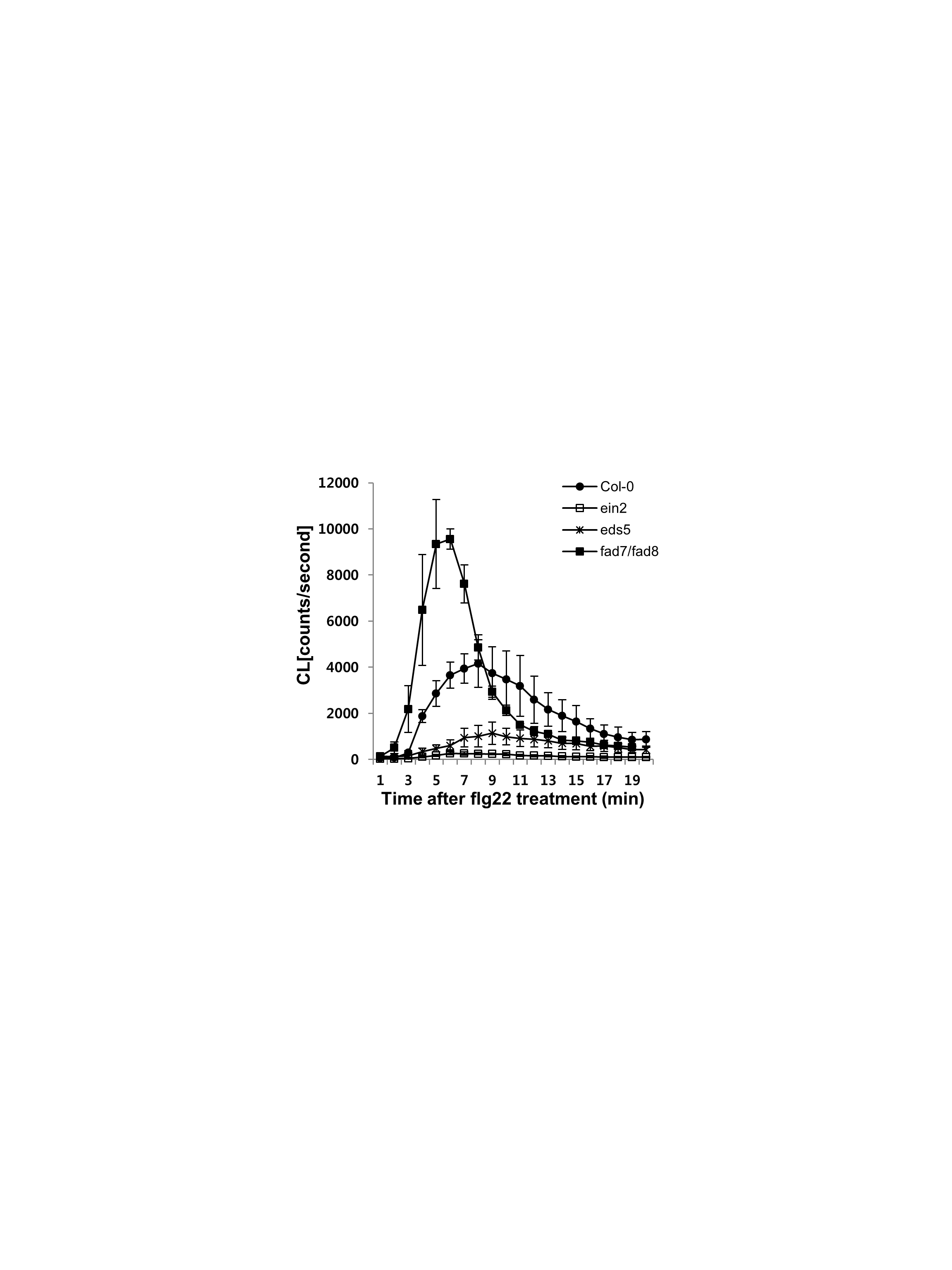

Supplement: Figure S1 — SA- and JA-signaling are required for the flg22-triggered oxidative burst. Flg22-induced ROS generation was monitored in liquid-grown intact seedlings of the indicated genotypes after treatment with 1 µM flg22. Error bars represent the SD from five independent samples (n = 10) and similar results were obtained in multiple independent experiments. (TIF) [file pone.0088951.s002.tif]

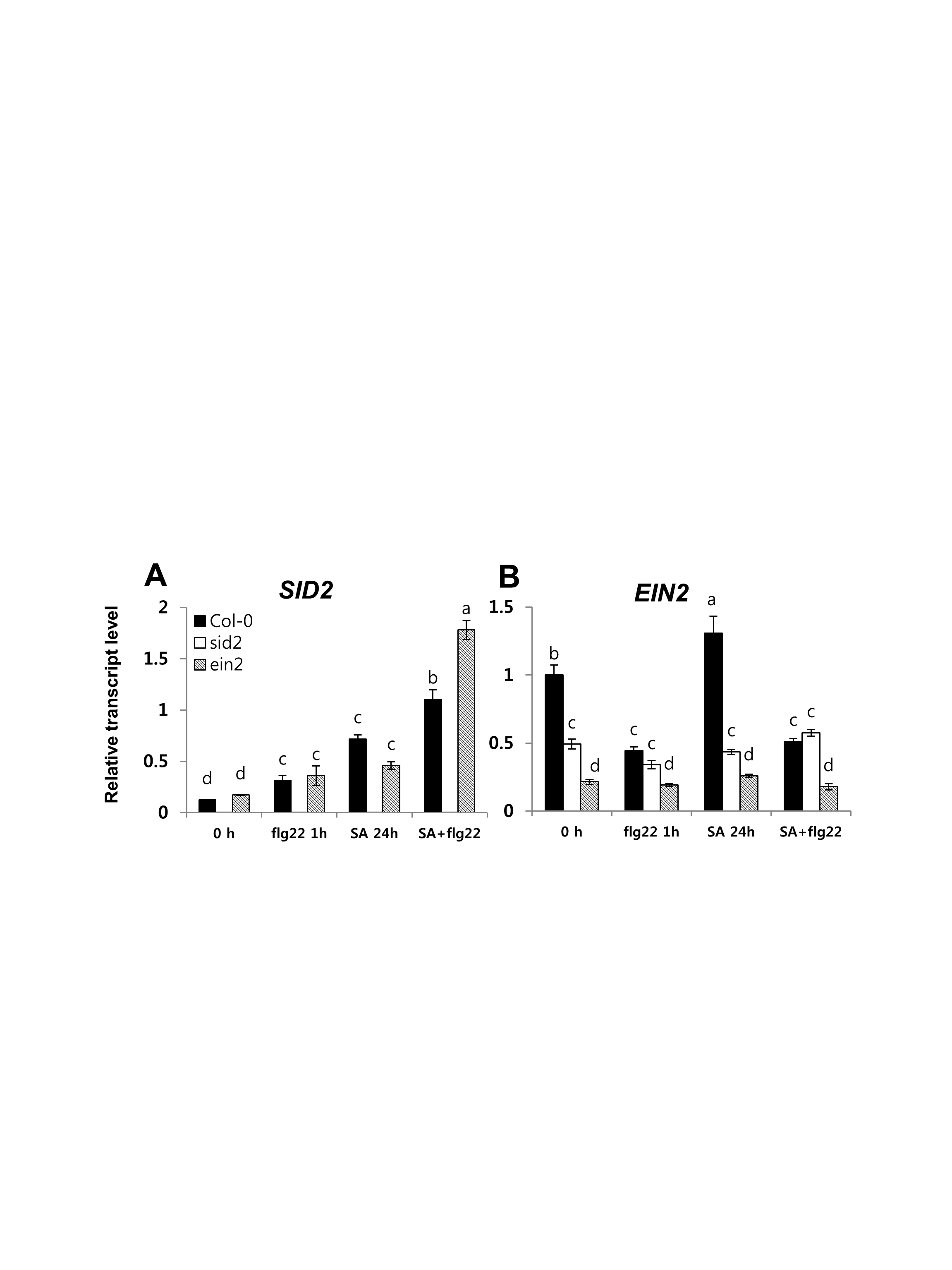

Supplement: Figure S2 — Down regulation of the ein2 gene in sid2 plants. For Quantitative RT-PCR analysis, 8-day-old seedlings were pre-treated with 100 µM of salicylic acid for 24 h and then incubated in 1 µM flg22 solution for 1 h. ACT2 [74] was used as a control. Data represent SD. All quantitative gene expression measurements were performed using technical triplicate and biological duplicates. Differential letter types indicated significant differences (α = 0.05) by one-way ANOVA and Tukey HSD test of comparisons between plant genotypes with individual treatment. (TIF) [file pone.0088951.s003.tif]

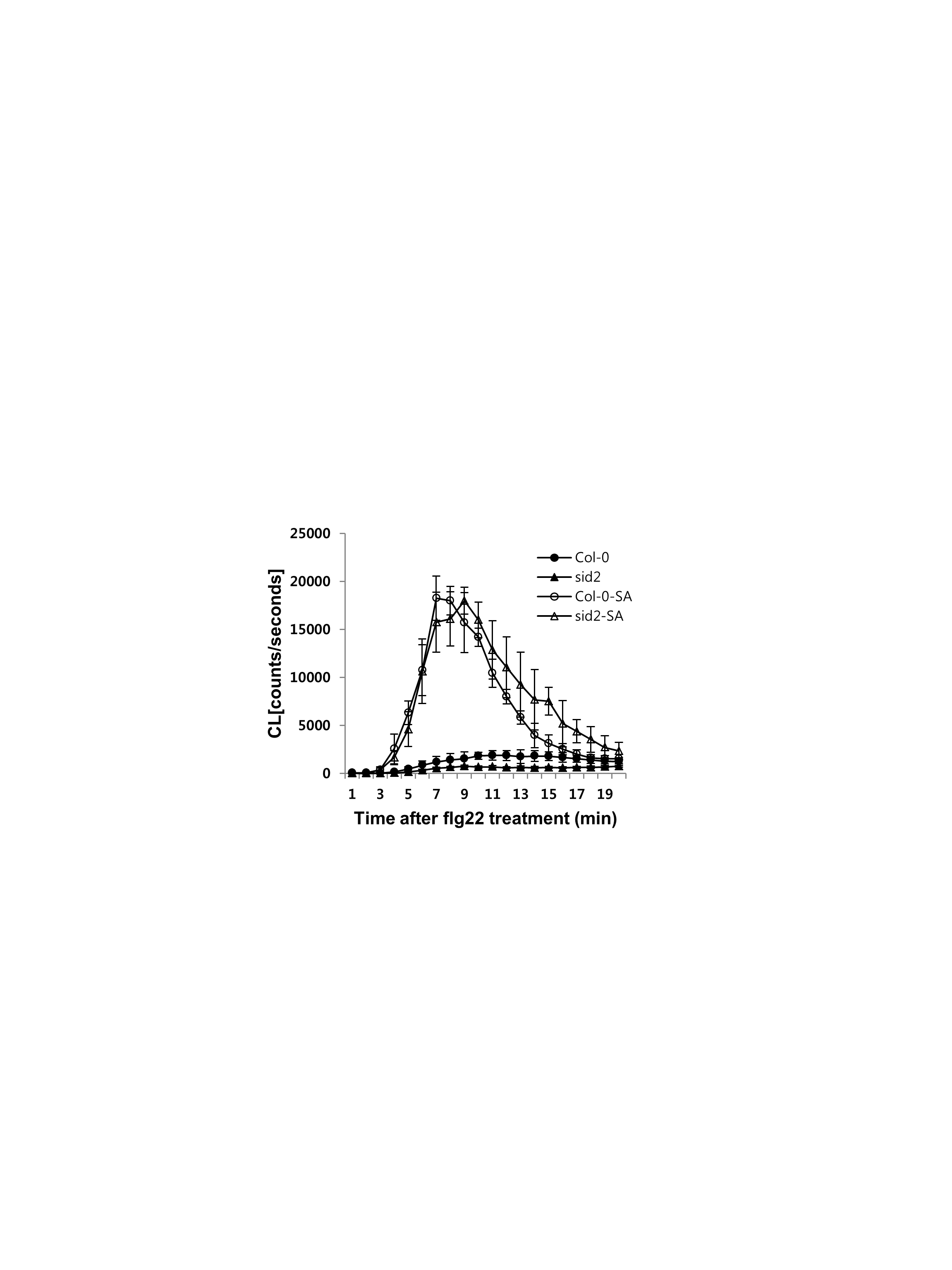

Supplement: Figure S3 — SA pretreatment reversed the suppressed flg22 response in sid2 mutants. For ROS measurement, 8-days-old seedlings were pretreated with 100 µM SA for 24 h and 1 µM flg22 was added at zero time. ACT2 was used as control. Error bars represent the SD of five independent samples (n = 10) and similar results were obtained in three independent experiments. (TIF) [file pone.0088951.s004.tif]

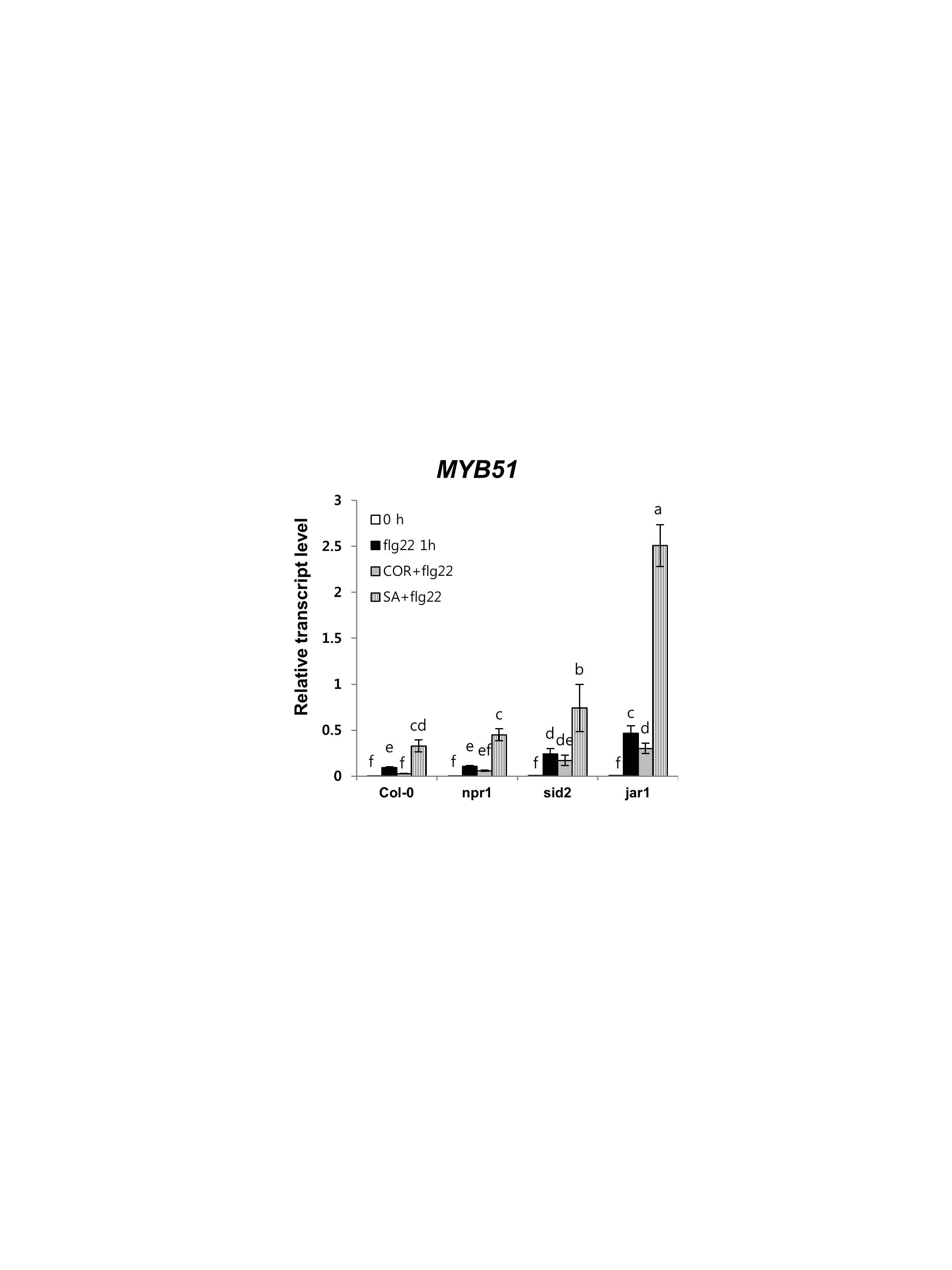

Supplement: Figure S4 — Effect of SA or COR pretreatment in flg22-induced MYB51 mRNA accumulation. Quantitative RT-PCR analysis of MYB51 gene expressions were measured in 8-day-old seedlings 1 h after treatment of 1 µM flg22. ACT2 was used as control. Data represent SD. All quantitative gene expression measurements were performed using technical triplicates and biological duplicates. Differential letter types indicated significant differences (α = 0.05) by one-way ANOVA and Tukey HSD test of comparisons between plant genotypes with individual treatment. (TIF) [file pone.0088951.s005.tif]

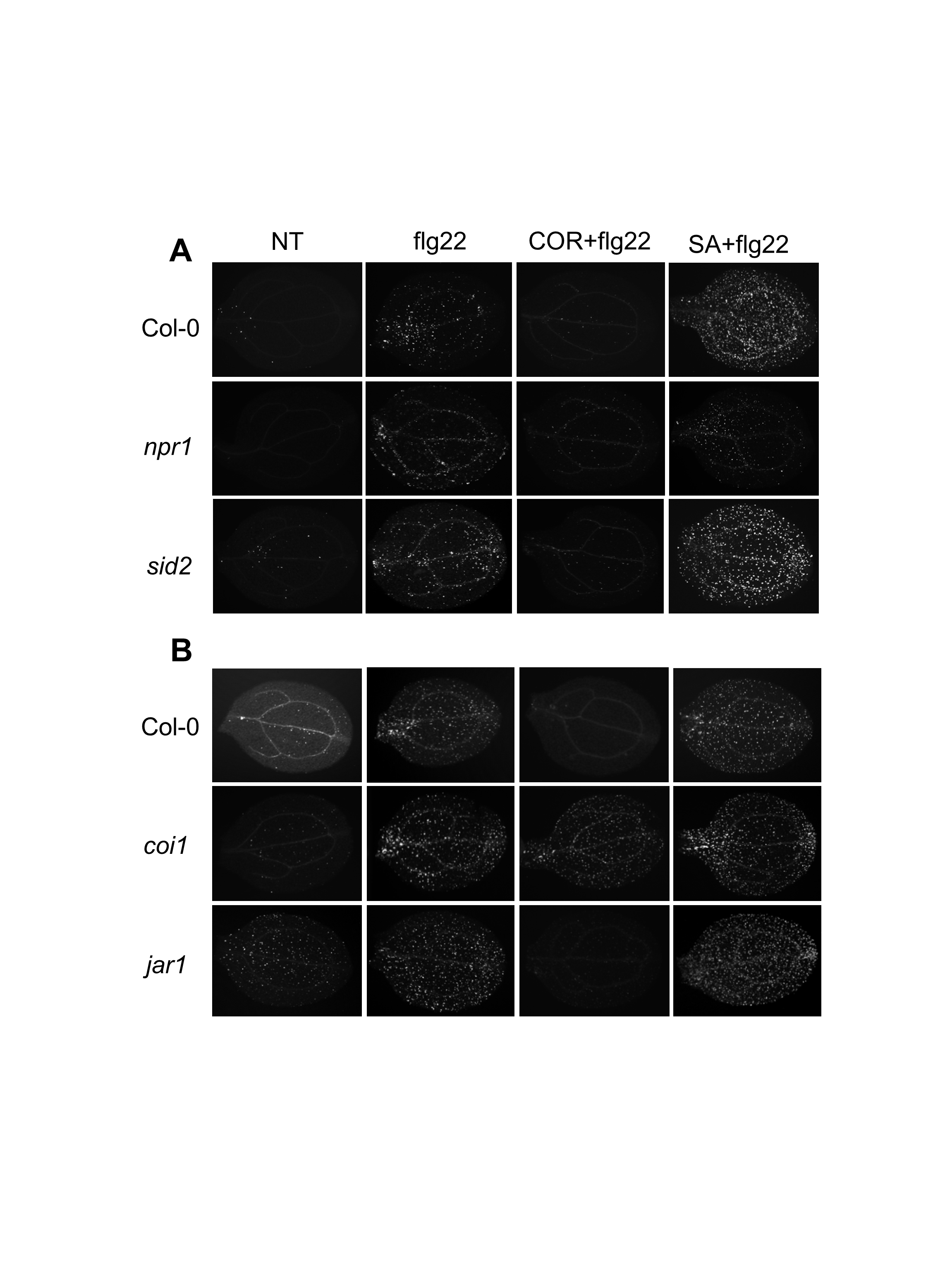

Supplement: Figure S5 — Effect of either SA or COR pretreatment on flg22-induced callose deposition. At 24 h post-treatment, cotyledons were stained with aniline blue. Fluorescence was observed with a NIKON AZ 100 M microscope. Representative images shown here came from eight leaves of eight independent plants, and similar results were obtained from two independent experiments. (TIF) [file pone.0088951.s006.tif]
